# *N*-phenyl-6-chloro-4-hydroxy-2-quinolone-3-carboxamides: Molecular Docking, Synthesis, and Biological Investigation as Anticancer Agents

**DOI:** 10.3390/molecules26010073

**Published:** 2020-12-25

**Authors:** Dima A. Sabbah, Rawan A. Haroon, Sanaa K. Bardaweel, Rima Hajjo, Kamal Sweidan

**Affiliations:** 1 Department of Pharmacy, Faculty of Pharmacy, Al-Zaytoonah University of Jordan, Amman 11733, Jordan; rawan21993.ra@gmail.com (R.A.H.); r.hajjo@zuj.edu.jo (R.H.); 2 Department of Pharmaceutical Sciences, School of Pharmacy, The University of Jordan, Amman 11942, Jordan; s.bardaweel@ju.edu.jo; 3 Department of Chemistry, The University of Jordan, Amman 11942, Jordan; k.sweidan@ju.edu.jo

**Keywords:** anticancer, colon cancer, PI3Kα, AKT, docking, quinolone-3-carboxamide

## Abstract

Cancer is a multifactorial disease and the second leading cause of death worldwide. Diverse factors induce carcinogenesis, such as diet, smoking, radiation, and genetic defects. The phosphatidylinositol 3-kinase (PI3Kα) has emerged as an attractive target for anticancer drug design. Eighteen derivatives of *N*-phenyl-6-chloro-4-hydroxy-2-quinolone-3-carboxamide were synthesized and characterized using FT-IR, NMR (^1^H and ^13^C), and high-resolution mass spectra (HRMS). The series exhibited distinct antiproliferative activity (IC_50_ µM) against human epithelial colorectal adenocarcinoma (Caco-2) and colon carcinoma (HCT-116) cell lines, respectively: compounds **16** (37.4, 8.9 µM), **18** (50.9, 3.3 µM), **19** (17.0, 5.3 µM), and **21** (18.9, 4.9 µM). The induced-fit docking (IFD) studies against PI3Kαs showed that the derivatives occupy the PI3Kα binding site and engage with key binding residues.

## 1. Introduction

The emergence of cancer cases continues to rise worldwide. Cancer, a group of diseases, is considered a prevalent health problem and cause of death. Cancer is uncontrolled cell proliferation accompanied with invasion to other body organs [1]. Multiple factors induce cancer occurrence, such as smoking, diet, radiation, viral infections, and genetic disorders [2,3,4,5]. Phosphatidylinositol 3-kinases (PI3Ks) catalyze the phosphorylation of phosphatidylinositol on the 3-OH group of inositol ring generating phosphatidylinositol 3,4,5 triphosphates (PIP_3_). PIP_3_ invoke downstream effectors and signaling proteins such as protein kinase B (AKT) inducing cell growth and multiplication [6,7].

The phosphatase and tensin homolog (PTEN) dephosphorylates the 3-OH group of PIP_3_ producing PIP_2_ and therefore antagonizes PI3Ks [7,8]. PI3Ks are organized into three classes (I, II, and III) according to their substrate interaction and protein sequence. Class IA PI3Ks accommodate PI3Kα, β, and δ isoforms encoded by their corresponding genes *PIK3CA*, *PIK3CB*, and *PIK3CD* [9]. Irregular activation of the PI3Kα/AKT signaling pathway has been observed in multiple human cancers [10]. The *PIK3CA* is catalyzed, over encrypted, and altered in human cancers [10]. Mutations of PI3Kα domains, the helical (E545K and E542K) and kinase (H1047R), are identified in breast, brain, GIT, and uterus cancers [11,12,13,14]. These carcinogenic transformations deform PI3Kα trajectories and thus in turn motivate researchers to design and develop selective PI3Kα inhibitors [13]. The ubiquity of PI3Kα and PTEN alterations in diverse human cancers highlights PI3Kα as an attractive target for anticancer drug design [15,16].

**Figure 1:**
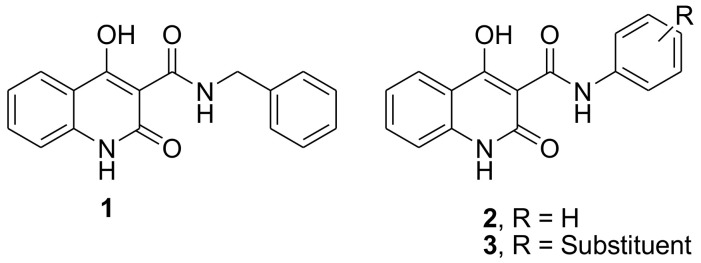
Chemical structures of **1, 2**, and **3**.

**Figure 2:**
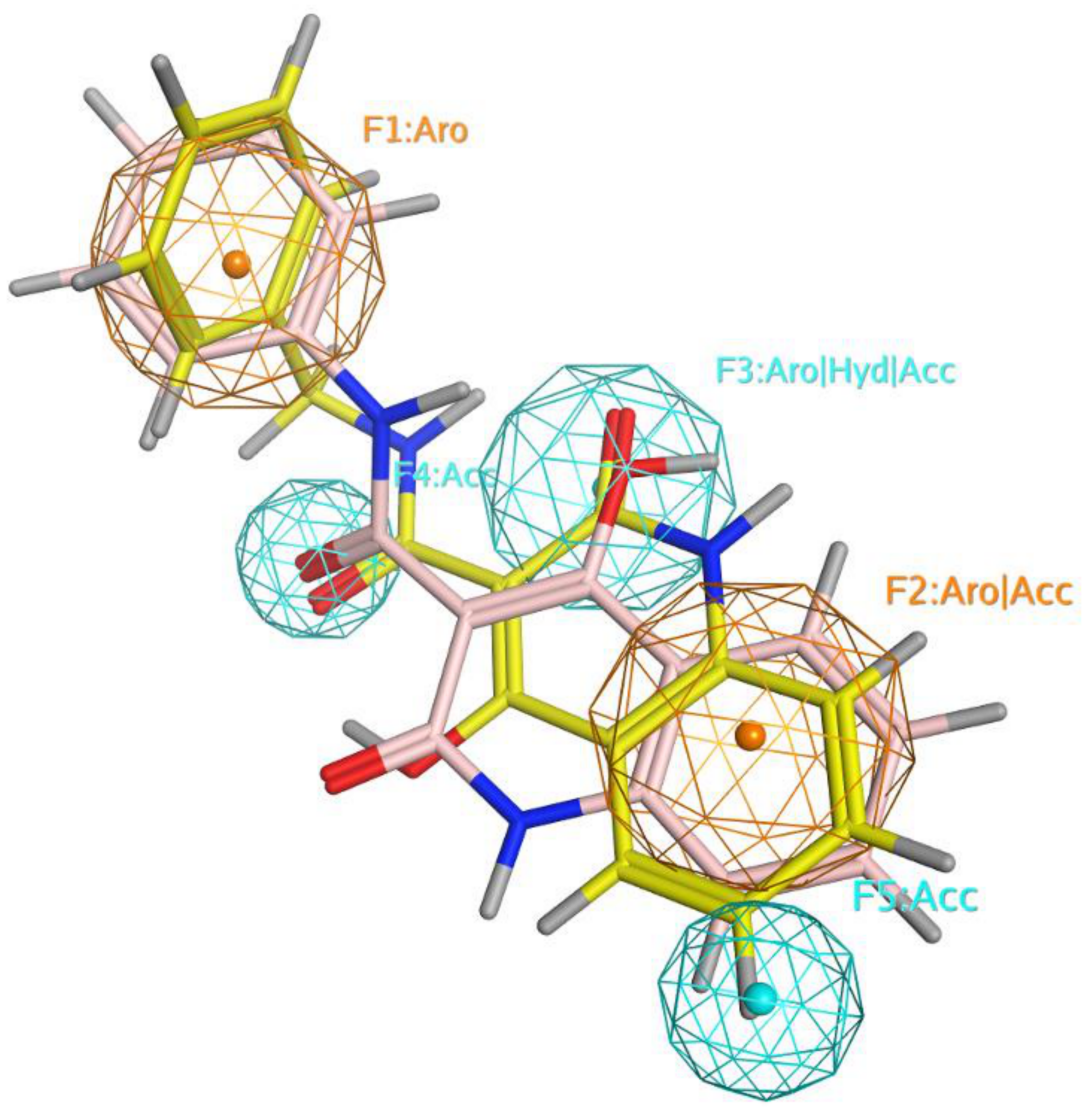
Phosphatidylinositol 3-kinase (PI3Kα) inhibitor pharmacophore model with compounds **1** (yellow color) and **2** (pink color). Picture captured by MOE [20].

Applying pharmacophore modeling and database screening against the national cancer institute (NCI) database [17], we previously revealed *N*-benzyl-4-hydroxy-2-quinolone -3- carboxamide (**1**) as a lead inhibitor against the wild-type (WT) and mutant (MUT) H1047R PI3Kα with IC_50_ values of 1.1 μM and 0.73 μM, respectively (Figure 1 and Figure 2) [18]. Furthermore, we identified the conformational changes of WT and MUT PI3Kα that might be pinpointed to develop selective inhibitors [19].

In addition, we determined the significant binding residues of PI3Kαs inhibitors by implementing molecular mechanics/generalized born surface area (MM/GBSA) procedure to calculate binding free energy for PI3Kα molecular dynamic (MD) extracted co-ordinates [21]. Besides, ceaseless lead optimization tactics disclosed *N*-phenyl-4-hydroxy-2-quinolone-3-carboxamide derivatives as selective (MUT) H1047R PI3Kα inhibitors illustrated by **2** [22]. Recently, we published a series of *N*-substituted- 4-hydroxy-2-quinolone-3-carboxamides that demolished human colon carcinoma (HCT116) cell line differentiation and promoted apoptosis by catalyzing caspase-3 activity and damaging cellular DNA represented by **3** [23]. Also, our continuous attempts released various core nucleus [24,25,26,27,28,29,30] targeting the kinase domain of PI3Kα.

In this respect, we designed and synthesized a series of *N*-phenyl-6-chloro-4-hydroxy-2-quinolone-3-carboxamides to explore the effect of introducing chloro functionality on the inhibitory activity and to clarify the structure–activity relationship (SAR) of the analogues. Biological testing of the verified compounds and pan PI3K inhibitor (LY294002) was probed in vitro against human epithelial colorectal adenocarcinoma (Caco-2) and human colon carcinoma (HCT-116) cell lines.

## 2. Results and Discussion

### 2.1. Chemistry

**Scheme 1:**
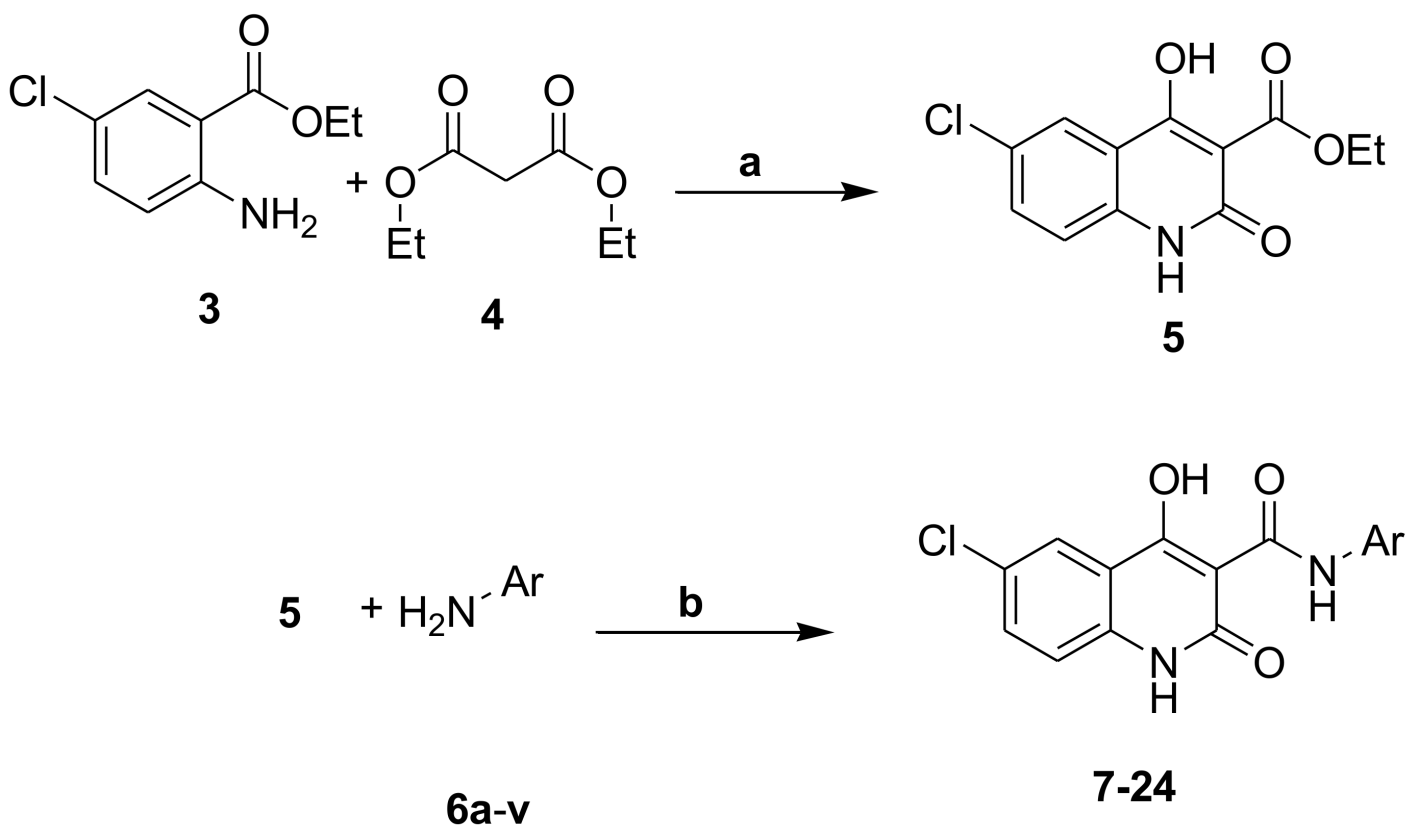
Conditions: (**a**): (i) NaOC_2_H_5_, DMSO, 145–150 °C, 72h. (ii) 0.3M HCl (**b**): (i) THF, DMF, 150 °C, 48–72h.

Verified compounds (**7–24**) were designed to investigate the effect of attaching different motifs at the carboxamide link of 6-chloro-4-hydroxy-quinolinone core structure and their inhibitory activity. Reaction of **3** and **4** in sodium ethoxide under reflux produced the core nucleus **5** with 88% yield, as represented in Scheme 1. Thin-layer chromatography (TLC) application was recruited to monitor the reaction progress; the absence of a spot belonging to **3** highlighted the completeness of the reaction.

Target compounds **7–27** have been generated by refluxing **5** with an excess of aromatic amine (ArNH_2_) using DMF (dimethylformamide.), as depicted in Table 1 and Scheme 1. The disappearance of a spot of compound **5** on the TLC plate suggested that the reaction was finished. The identity of the chemical backbones was confirmed using IR, NMR and high-resolution mass spectra (HRMS). The elaborate experimental and spectroscopic data agree with the prospective structures.

### 2.2. Biological Evaluation of the Synthesized Compounds

In order to evaluate the antiproliferative activity of the prospective compounds (**5**, **7–24**) against the kinase domain of PI3Kα, we examined their inhibitory activity in human epithelial colorectal adenocarcinoma (Caco-2) and the human colon cancer (HCT-116) cell line as shown in (Table 2). The epithelial colorectal adenocarcinoma (Caco-2) encodes the wild-type (WT) PI3Kα [31,32,33]. The malignant (HCT-116) carcinoma expresses both wild-type (WT) and mutant (MUT) (H1047R) PI3Kα [34]. Therefore, the difference in activity against Caco-2 and HCT-116 corresponds to MUT (H1047R) PI3Kα.

Screening against Caco-2 showed that compounds tailored with *o*-CF_3_ (**8**) or *m*-CF_3_ (**9**) provoke the activity suggesting that hydrophobic and/or H-bond interaction mediate(s) ligand/PI3Kα complex formation. Similarly, the antiproliferative activity of *p*-CF_3_ (**10**) implies that hydrophobic and/or H-bond interaction guides (s) ligand/PI3Kα interaction at *p*-position.

The antiproliferative activity of *p*-OCH_3_ (**11**) and *p*-CH_3_ (**12**) proclaims that oxygen atom places the -CH_3_ deeply in the binding pocket close to the hydrophobic lining, thus inducing the activity. Additionally, the activity of *p*-CF_3_ (**10**) and *p*-CH_3_ (**12**) indicates that H-bond drives ligand/PI3Kα interaction on *p*-position. Interestingly, elongation of the carboxamide side chain by one carbon exemplified by **7** enhances the activity assuming that the aromatic motif is buried deeply in the hydrophobic site. This result presumes that elongation of the carboxamide side chain (**7**) or tailoring the phenyl by –OCH_3_ (**11**) situates the hydrophobic motif in the hydrophobic cleft.

Tailoring the carboxamide side chain with substituted benzoic acid moiety (**13**, **14**, and **15**) reveals that H-bond mediates ligand/PI3Kα binding on *o*-and *m*-positions and thus in turn provides an explanation for the activity of **8** and **9**. Furthermore, the activity of **14** suggests that the carboxylic group on *o*-position might be involved in intra H-bond generating a conformer that better orientates the analogue in the binding domain. Also, the activity of **13**, **14**, and **15** anticipates the substantial effect of choloro motif on better accommodation in the binding cleft. Introducing a heterocyclic group on the side chain of carboxamide illustrated by (**16** and **17**) confirms the significance of the aromatic or heterocyclic group for better activity. Additionally, the nitrogen atom in pyridine provides H-bond acceptor and promotes water solubility and polarity.

The activity of **18** affirms the importance of substituted benzoic acid motif and declares that the ionized carboxylate anion might impede cell membrane permeability. Additionally, the activity of **18** provides a clue for the beneficial influence of choloro group on cell membrane permeability as exemplified by **13**, **14**, and **15**. Attaching a fluorinated benzene moiety on carboxamide side chain (**19**, **20**, and **21**) induces the activity interrogating that the fluoro moiety on *o*-, *m*-, and *p*-positions properly accommodates the binding site due to its small size. Introducing the methylated benzoic acid motif (**22** and **23**) elicits the activity inferring that hydrophobic interaction mediates ligand/PI3Kα interaction on *o*-and *m*-positions corresponding to -COOH. Contrarily, methoxylated benzoic acid moiety (**24**) induces the activity implying that H-bond and/or hydrophobic interaction on *p*-position, relatively to-COOH, drives ligand/PI3Kα binding. 

Screening against HCT-116 revealed that elongating the carboxamide side chain (**7**) induces the inhibitory activity inferring better accommodating in the binding domain. The biological data of *o*-CF_3_ (**8**), *m*-CF_3_ (**9**), and *p*-CF_3_ (**10**) declare that hydrophobic and/or H-bond mediates ligand/PI3Kα interaction on *o*-position. The inhibitory activity of *p*-OCH_3_ (**11**) anticipates that the oxygen atom might push the –CH_3_ properly in the binding domain. Furthermore, the activity of *p*-CH_3_ (**12**) confirms the significance of oxygen atom and provides further explanation for the activity of *p*-OCH_3_ (**11**). Additionally, the inhibitory activity of *p*-CF_3_ (**10**) and *p*-CH_3_ (**12**) elucidates the importance of H-bonding at the *p*-position. Attaching a substituted benzoic acid moiety on carboxamide side chain (**13**, **14**, and **15)** shows that the *p*-chloro moiety provokes the activity anticipating a favored conformation is mediated by intra-H-bond between -COOH and the carboxamide motif. Tailoring the carboxamide side chain with pyridine-4-yl (**16**) induces the activity inferring that H-bond and/or ionic interaction mediates **16**/PI3Kα complex formation. Additionally, the activity of pyridine-3-yl (**17**) affirms the significance of pyridine-4-yl on inducing the inhibitory activity. 

Attaching 3-benzoic acid moiety (**18**) elicits the activity suggesting that H-bond and/or ionic interaction mediates ligand/PI3Kα interaction. Incorporating a fluorinated benzene moiety (**19**, **20**, and **21)** shows that the *o*-and *p*-fluoro analogs mediate H-bond with key binding residues. Attaching a methylated benzoic acid motif (**22** and **23**) suggests that the intra-H bond offered by –COOH and the carboxamide motif generates a conformer that impedes their proper orientation in the binding domain. The activity of methoxylated benzoic acid moiety (**24**) declares that the oxygen atom might push -CH_3_ in the binding sites and the existence of –COOH might provide an intra-H-bond that orientates **24** properly in the binding site. Furthermore, the difference in the biological activity of **16**, **18**, **19**, **21**, and **24** against HCT-116 proclaims that selectivity is attained. Finally, the activity of **5** against Caco-2 and HCT-116 cell lines emphasizes the significance of substituted carboxamide motif.

Noting that the probed cell lines express both WT and MUT PI3Kαs, separating either enzyme is strongly advised to validate the suppressive activity against isolated PI3Kα. Future confirmation should be accomplished by silencing either gene to explore the antiproliferative mechanism against each PI3Kα.

**Figure 3:**
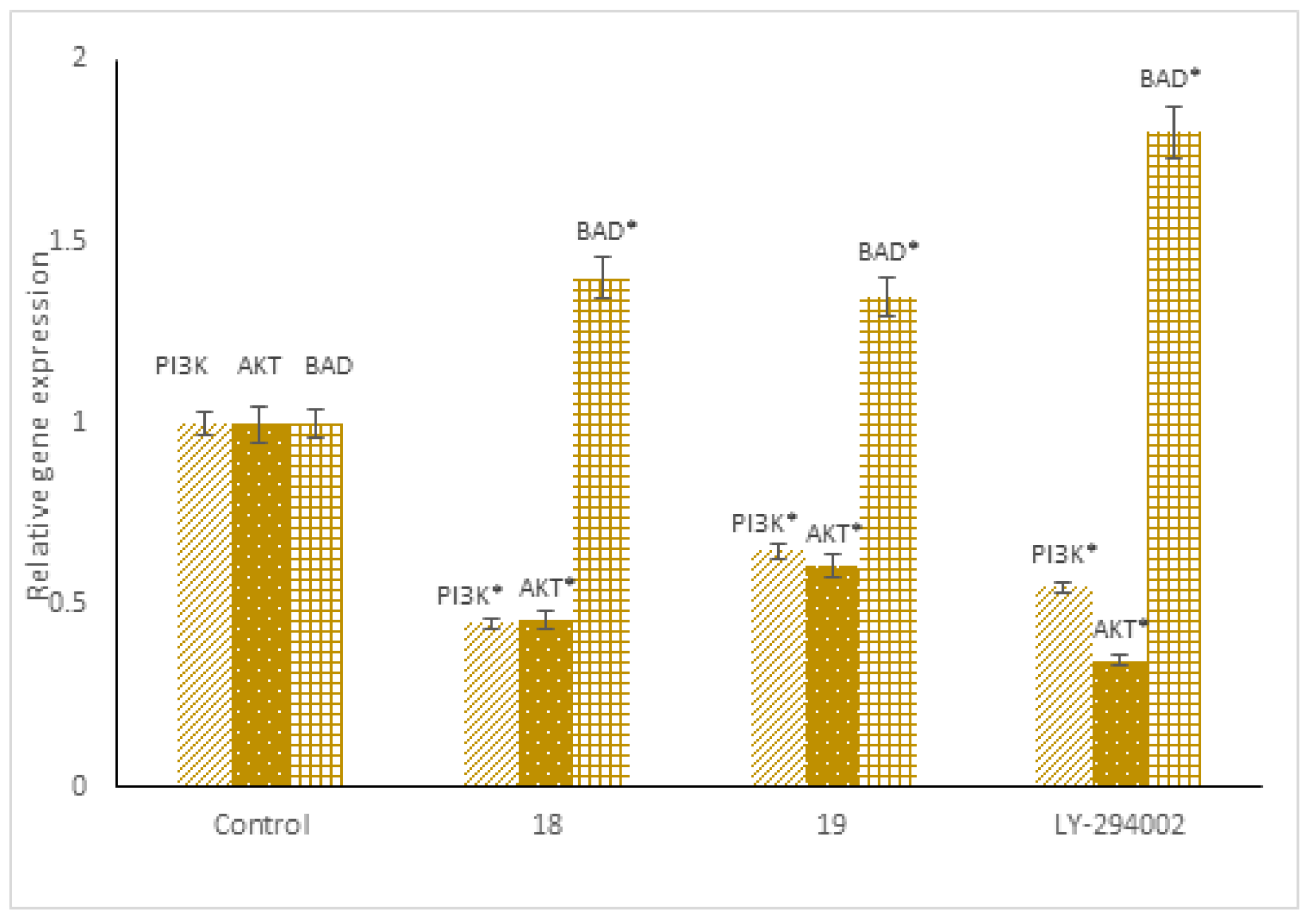
Relative gene expression of PI3K, AKT, and BAD in HCT116 cells after treatment with Compounds **18** and **19** and the positive control LY-294002. Bars represent mean values ± SEM (lines) of three replicates. Asterisks represent significant change relative to the negative control (* *p* < 0.05).

The effects of compounds (**18** and **19**) on the PI3K/AKT signaling pathway were investigated using qPCR. The relative gene expression of PI3K, AKT, and BAD (BCL2 Associated Agonist of Cell Death) was significantly changed by treatment with either compound (0.5 µM) in a manner consistent with the positive control treatment (0.5 μM) (Figure 3). A substantial reduction in PI3K and AKT gene expression was observed upon treatment with **18, 19,** or the positive control LY-294002. On the other hand, the expression of the pro-apoptotic gene BAD was significantly increased in treated cells (for all tested compound including the positive control) when compared to the negative control where there was no treatment applied.

### 2.3. Computational Studies

#### 2.3.1. Molecular Docking

**Figure 4:**
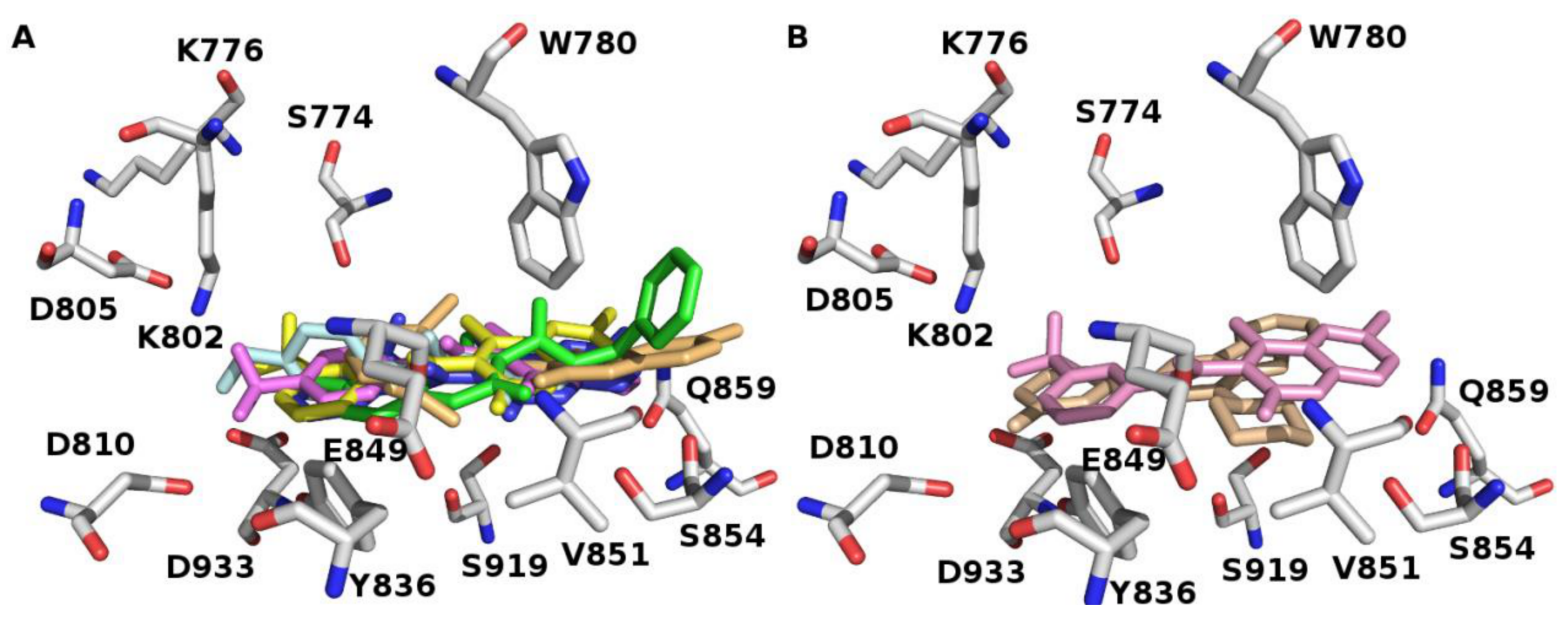
Kinase domain of (**A**) 2RD0 accommodating the IF docked geometries of some of the verified molecules and (**B**) superposing of the IF docked pose of **10** (pink color) and native pose of (X6K) (gold color). Some of the key binding residues are presented and H atoms are invisible for clarification. Picture made by PYMOL [38].

In order to discuss the anticancer activity of the synthesized compounds (**5** and **7–24**) in human colon carcinoma (HCT-116) and (Caco-2) cell lines, we recruited the co-ordinates of native (PDB ID: 2RD0) [9] and mutant H1047R (PDB ID: 3HHM) [35] PI3Kα to determine the structural basis of PI3Kα/ligand interaction. In order to probe the binding interaction of PI3Kα and the verified compounds (**5** and **7–24**) in the PI3Kα kinase domain, we accomplished induced-fit docking (IFD) [36,37,38] studies against 2RD0 and 3HHM. IFD data demonstrate that **5** and **7–24** accommodate PI3Kαs kinase domains (Figure 4A). Indeed, the docked geometry of **10** overlays the co-ordinate of X6K in the PI3Kα kinase site (PDB ID: 4L23) [39] (Figure 4B).

**Figure 5:**
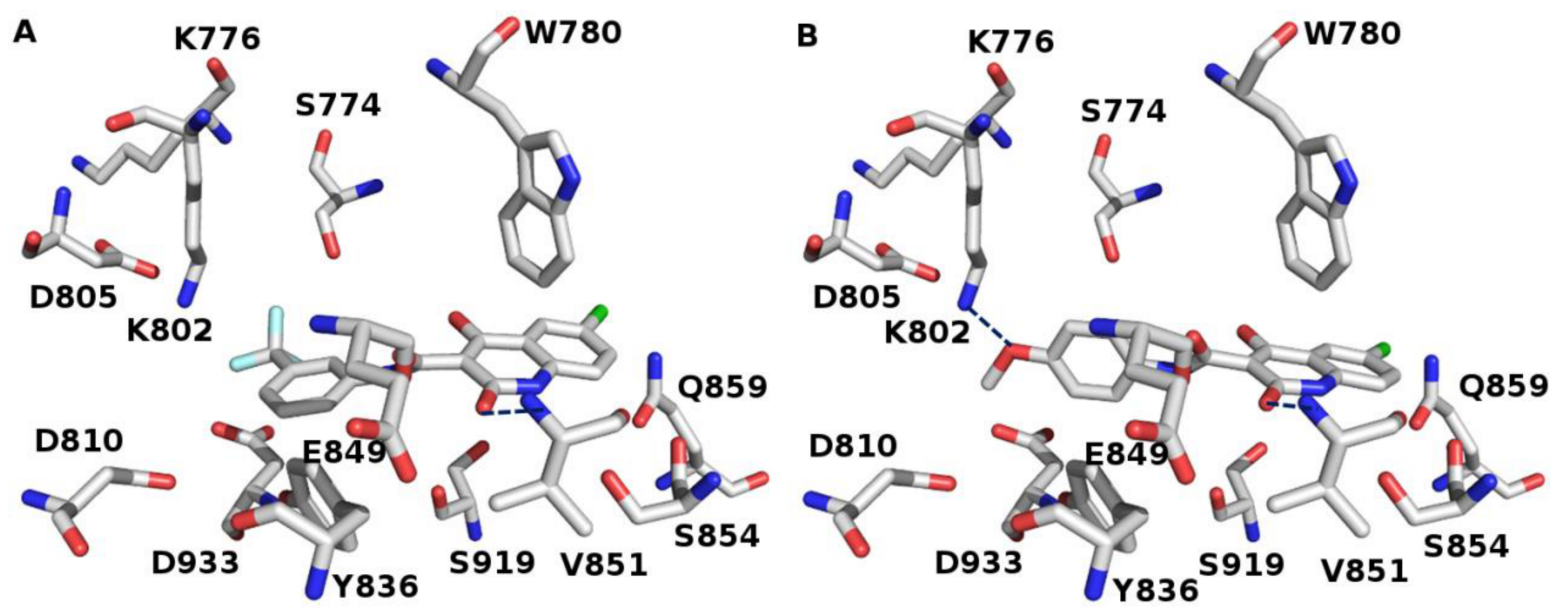
Binding poses of (**A**) **9** and (**B**) **11** in the 2RD0 kinase site. The H-Bond is represented as a blue dotted line. H atoms are hidden for clarification. Picture made by PYMOL [38].

The IFD approach explores the structural changes in proteins in this way: ligands are docked to a protein’s binding domain by Glide docking [36,37,38] and the top ligand conformation is minimized together with PI3Kα binding cleft by the Prime algorithm [38]. Next, a redocking protocol is performed against the minimized PI3Kα. Accordingly, protein flexibility is investigated during the docking procedure. The synthesized core structures make H-bond with S774, A775, K776, W780, E798, K802, D810, Y836, V851, N853, S854, N920, and D933 (Table 3) (Figure 5). Additionally, other computational [19,21,22,23,24,25,28,29,40] and experimental studies [9] highlighted the importance of these amino acids in PI3Kα/ligand complex formation.

Remarkably, **5**, **7–24** showed an affinity against both WT and MUT (H1047R) PI3Kα. Fortunately, higher binding affinity against 3HHM was recorded for **12** and **15**. Around 2 Kcal difference in binding energy against PI3Kαs might predict that this series is mutant selective. Furthermore, the dominance of S774 and K802 in ligand/PI3Kα complex formation infers that the scaffold might be PI3Kα-selective inhibitors [19].

**Figure 6:**
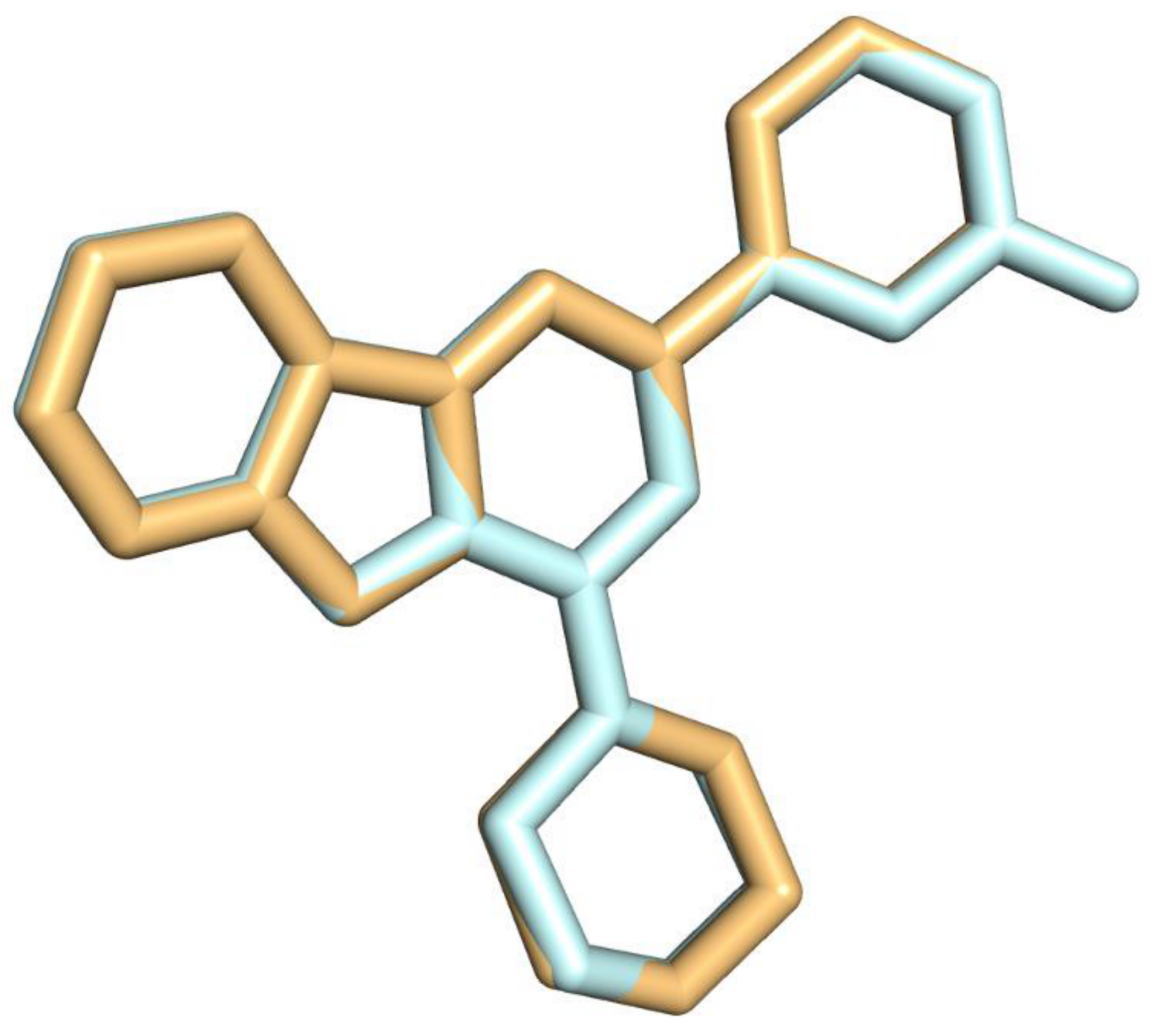
Superposition of the IFdocked X6K pose (blue color) and its native conformation (gold color) in 4L23. Picture made by PYMOL.

To evaluate the execution of the IFD algorithm, we compared the docked pose of X6K in WT PI3Kα (PDB ID: 4L23) [39] to its native geometry in the crystal assembly. Figure 6 demonstrates the overlaying of the IFD-extracted X6K conformation and its native geometry in 4L23. The RMSD for heavy atoms of X6K between IFD-extracted docked conformation and the native pose was 0.169 Å. The result infers that IFD can determine the native geometry in crystal co-ordinates and prosperously anticipate the ligand binding pose.

**Figure 7:**
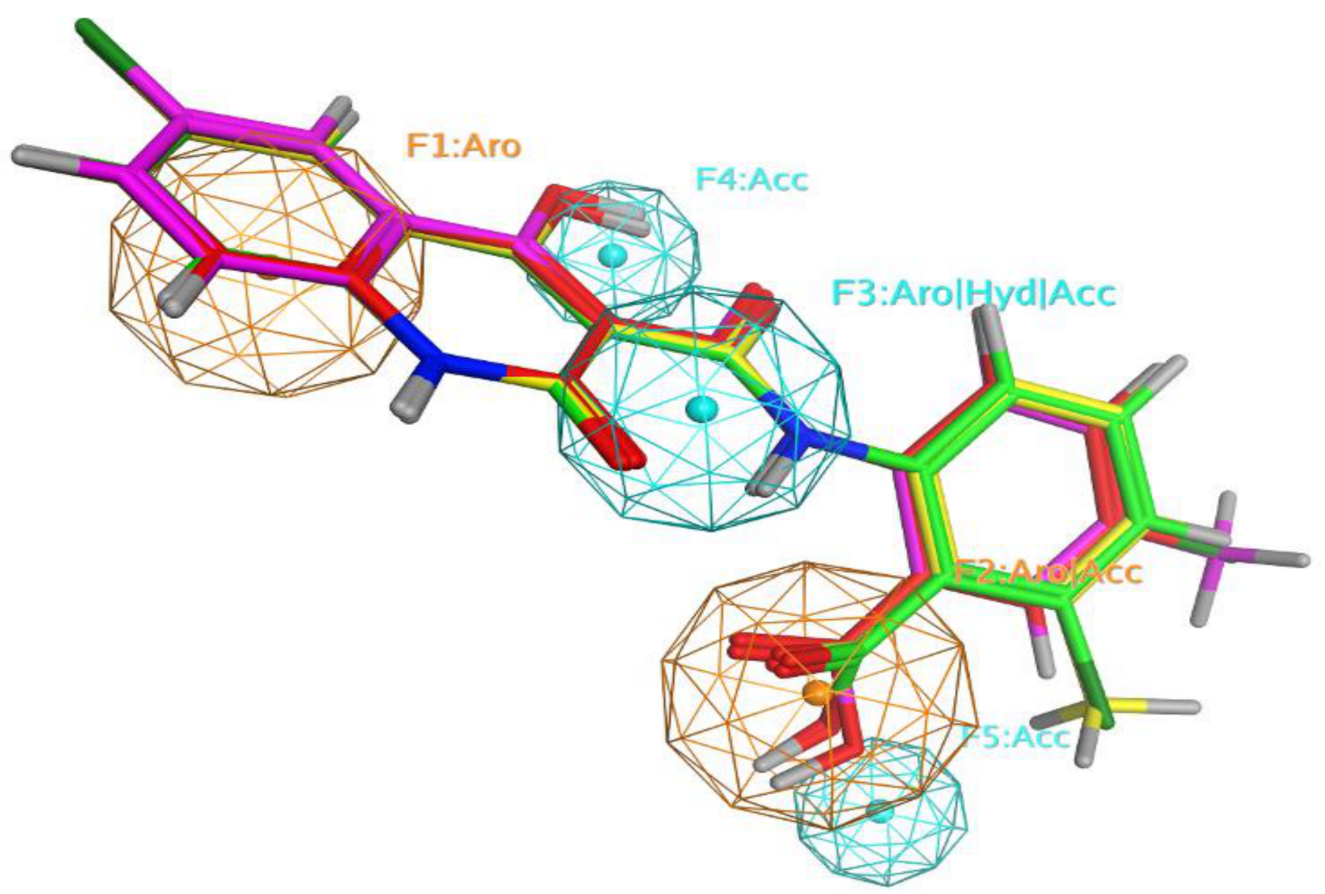
PI3Kα inhibitors pharmacophore model with **14** (red color), **15** (green color), **22** (pink color), and **23** (yellow color).

In order to inspect the binding groups of **7–24**, we screened them against a reported pharmacophore model of active PI3Kα inhibitors [18]. The core structure of **7–24** fit the functionalities of active PI3Kα inhibitors (Figure 7), illustrated as (F1) indicating one aromatic ring; (F2) one aromatic or H-bond acceptor; (F3) one aromatic or hydrophobic or H-bond acceptor; and either (F4) or (F5) one H-bond acceptor. The result interrogates the affinity of this scaffold against the PI3Kα binding site. Additionally, the occupation of **7–24** in the kinase domain predicts their possible PI3Kα antiproliferative activity.

#### 2.3.2. Descriptor Analysis

**Figure 8:**
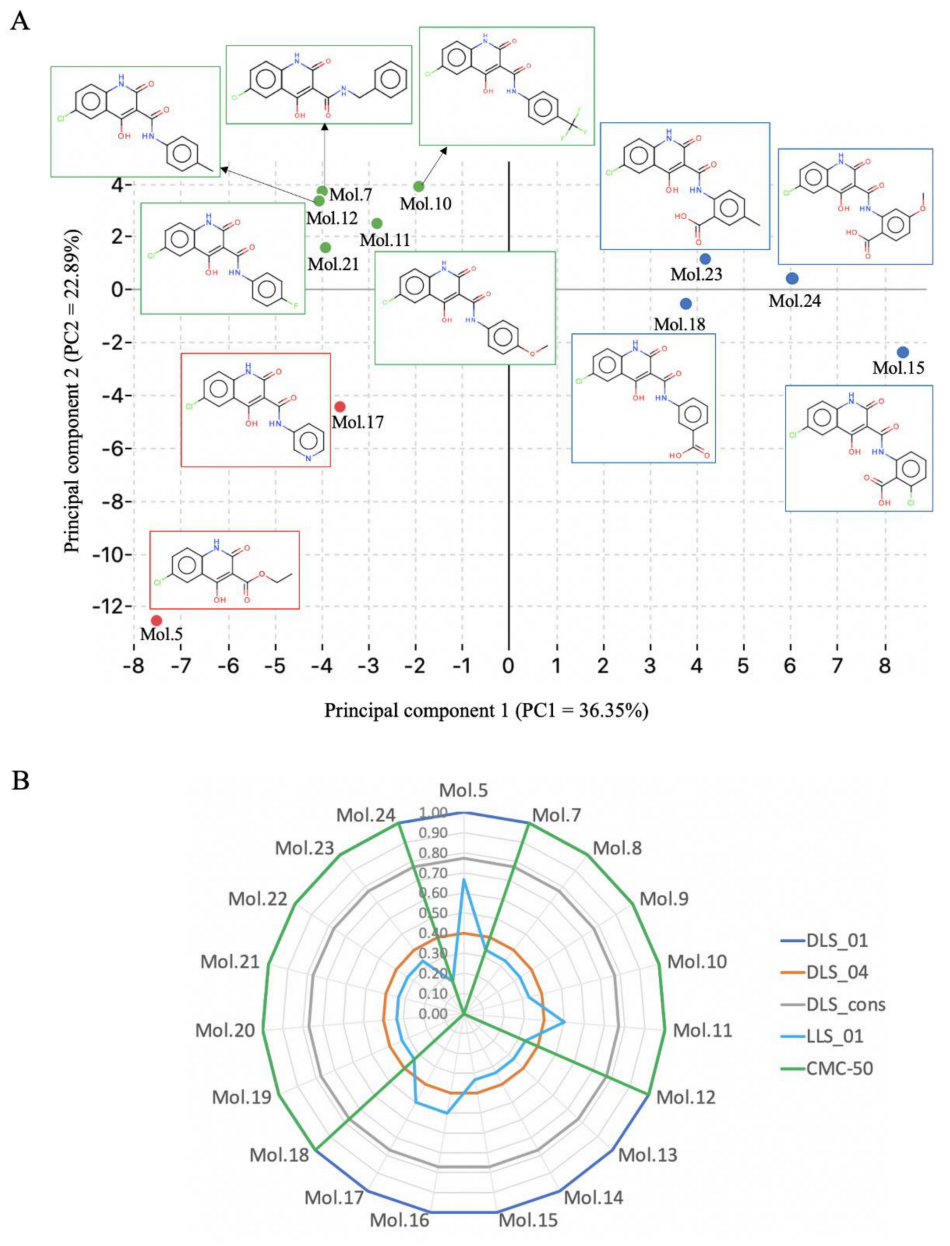
Molecular descriptor analysis. (**A**) A graphical 2D representation of the principal component analysis of molecular descriptors. PC1 explaining 36.35% of the variation in descriptor values is on the x-axis, and PC2 explaining 22.89% of the variation in descriptor values is on the y-axis. Descriptor used for the analysis belong two major categories of molecular descriptors: (1) drug-like indices; (2) molecular properties and P_VSA-like descriptors. (**B**) Radar plot of a representative set of 5 molecular drug-like and lead-like molecular descriptors that showed variability for the same compound.

Sixty-seven molecular descriptors comprising two main categories of alvaDecs [41] (i.e., drug-like indices, molecular properties and P_VSA-like descriptors) were calculated for molecules Mol.5–Mol.24. All descriptor values were then analyzed using principal component analysis. Our principal component analysis reveals that our compounds clustered into three main groups in the 3-D space formed by the first three principle components for the descriptor variance (Figure 8A). All calculated descriptors for all analyzed molecules are reported in Supplementary Table S1. Our analysis further revealed that our synthesized molecules differ in their lead-like and some drug-like properties as shown in Figure 8B.

## 3. Material and Methods

### 3.1. Chemistry

Chemical reagents and solvents were of a highly purified grade and used right away lacking any purification technique. Reagents were bought from Alpha-Chemika (Mumbai, India), Acros Organics (Fair Lawn, NJ, USA), Sigma-Aldrich (St. Louis, MI, USA), and Scharlau Company (Barcelona, Spain). Rota vapor (1) model R-215 (Buchi, Switzerland) attached to vacuum pump model v-700 and water bath B-491 were recruited to evaporate ordinary solvents and vacuum controller v-855 was used to evaporate high boiling solvents such as DMSO and DMF.

Melting points (MP) were recorded by Gallenkamp melting point apparatus. Hot plate and magnetic stirrer were delivered by vision scientific CO, LTD USA. Thin-layer chromatography (TLC) was conducted on 20 × 20 cm^2^ with layer thickness 0.2 mm aluminum cards pre-coated with fluorescent silica gel GF254 DC (Fluka analytical, Buchs, Switzerland) and detected by UV light indicator (at 254 and/or 360 nm).

Infrared (IR) spectra were recorded as, KBr discs, with a Shimadzu IR Affinity FTIR spectrophotometer. Nuclear magnetic resonance (NMR) ^1^H- and ^13^C-NMR spectra were measured on Bruker NanoBay 400 MHz spectrophotometer (the Hashemite University, Zarqa, Jordan). Chemical shifts were stated in *δ* (ppm) using TMS as an internal reference. High-resolution mass spectra (HRMS) were measured using a Bruker APEX-IV (7 Tesla) instrument. External calibration was performed using an arginine cluster at a mass range of *m/z* 175–871.

### 3.2. Synthesis of Target Compounds

#### 3.2.1. Ethyl 6-chloro-4-hydroxy-2-quinolone 3-carboxylate (5)

A mixture of ethyl 2-amino-5-chloro benzoate (**3**) (6.00 g, 30.06 mmol), an excess amount of diethylmalonate (**4**) (48.10 mL, 300.60 mmol), and sodium ethoxide (6.25 g, 91.80 mmol) in 25 mL DMSO was prepared and refluxed for 96 h. Completion of the reaction was observed by the absence of a spot of compound **3** on TLC. The generated solution was placed in an ice bath and then acidified with 0.3 M HCl reaching pH range of 4.5–5.0. A solid residue was formed, washed with water followed by MeOH (10 mL), and then dried in a vacuum oven at 70 °C for 1 h.

A beige powder; yield: 87.8%; R_ƒ_ = 0.64 (CHCl_3_: MeOH- 9.5: 0.5); m.p: 200–201 °C; ^1^H-NMR (400 MHz, DMSO-*d*_6+_NaOD): δ = 3.41–3.53 (m, 3H, -C*H*_3_), 4.06–4.10 (m, 2H, -C*H*_2_), 6.88 (d, *J* = 8.0 Hz, 1H, Ar-*H*), 6.97 (d, *J* = 8.0 Hz, 1H, Ar-*H*), 7.71 (s, 1H, Ar-*H*) ppm; ^13^C-NMR (100 MHz, DMSO-*d*_6_): δ = 15.0 (1C), 56.4 (1C), 102.0 (1C), 118.9 (1C), 124.1(1C), 124.2 (2C), 125.4 (1C), 127.9 (1C), 149.8 (1C), 172.9 (2C) ppm; IR (KBr disc): 3014, 2958, 2920, 2846, 1672, 1643, 1477, 1413, 1381, 1325 cm^−1^. High-resolution mass spectrum, *m/z*: calculated 268.03766 for C_12_H_11_ClNO_4_ [M + H]^+^; found 268.03786.

#### 3.2.2. *N*-benzyl 6-chloro-4-hydroxy-2-quinolone-3-carboxamide (7)

A mixture of ethyl 6-chloro-4-hydroxy-2-quinolone-3-carboxylate (**5**) (1.00 g, 3.74 mmol) and benzyl amine (**6a**) (1.20 g, 11.20 mmol) was prepared in THF (25 mL). A few drops of DMF were added to the solution and refluxed for 72 h. Reaction progress was monitored by the absence of a spot of **5** on TLC and a precipitate appearance at RT. A solid residue was formed, washed with H_2_O, MeOH, and THF, and then dried in a vacuum oven at 70 °C for 1 h.

A white powder; yield: 52.9%; R_ƒ_ = 0.8 (CHCl_3_: MeOH- 9.5:0.5); m.p: 360–362 °C; ^1^H-NMR (400 MHz, DMSO-*d*_6+_NaOD): δ = 3.53 (s, 2H, -C*H*_2_), 6.94 (d, *J* = 8.4 Hz 2H, Ar-*H*), 7.04–7.07 (m, 2H, Ar-*H*), 7.44–7.45 (m, 1H, Ar-*H*), 7.57 (d, *J* = 7.2 Hz 1H, Ar-*H*), 7.78 (s, 1H, Ar-*H*), 8.41 (d, *J* = 7.6 Hz, 1H, Ar-*H*) ppm; ^13^C-NMR (100 MHz, DMSO-*d*_6_): δ = 100.5 (1C), 119.4 (1C), 121.5 (1C), 124.4 (1C), 124.5 (1C), 125.7 (2C), 125.8 (1C), 132.4 (2C), 139.6 (1C), 149.6 (2C), 170.1 (2C), 174.0 (1C), 176.8 (1C) ppm; IR (KBr disc) 3192, 3078, 2918, 1666, 1633, 1458, 1415, 1371, 1323, 1282 cm^−1^. High-resolution mass spectrum, *m/z*: calculated 329.06930 for C_17_H_14_ClN_2_O_3_ [M + H]^+^; found 329.06984.

#### 3.2.3. *N*-(2-(trifluoromethyl) phenyl)-6-chloro-4-hydroxy-2-quinolone-3-carboxamide (**8**)

A mixture of ethyl 6-chloro-4-hydroxy-2-quinolone-3-carboxylate (**5**) (1.00 g, 3.74 mmol) and 2-trifluromethyl aniline (**6b**) (1.80 g, 11.20 mmol) was prepared in THF (25 mL). A few drops of DMF were added to the solution and refluxed for 72 h. Reaction progress was monitored by the absence of a spot of **5** on TLC and a precipitate appearance at RT. A solid residue was formed, washed with H_2_O, MeOH, and THF, and then dried in a vacuum oven at 70 °C for 1 h.

A white powder; yield: 55.6%, R_ƒ_ = 0.78 (CHCl_3_: MeOH- 9.5:0.5); m.p: 294–296 °C; ^1^H-NMR (400 MHz, DMSO-*d*_6+_NaOD): δ = 6.94–6.96 (m, 1H, Ar-*H*), 7.05–7.08 (m, 1H, Ar-*H*), 7.21 (d, *J* = 8.0 Hz, 1H, Ar-*H*), 7.4 (t, *J* = 8.0, 4.0 Hz, 1H, Ar-*H*), 7.76–7.81 (m, 1H, Ar-*H*), 8.25 (s, 1H, Ar-*H*) ppm; ^13^C-NMR (100 MHz, DMSO-*d*_6_): δ = 100.8 (1C), 119.7 (1C), 120.9 (1C), 123.1 (2C), 123.6 (1C), 124.3 (2C), 124.4 (1C), 124.7 (1C), 126.3 (1C), 127.7 (1C), 129.9 (1C), 149.3 (1C), 170.2 (1C), 173.7 (1C), 176.6 (1C) ppm; IR (KBr disc): 3171, 3074, 3018, 1660, 1570, 1458, 1417, 1375, 1332 cm^−1^. High-resolution mass spectrum, *m/z*: calculated 383.04103 for C_17_H_11_ClF_3_N_2_O_3_ [M + H]^+^; found 383.04151.

#### 3.2.4. *N*-(3-(trifluoromethyl)phenyl)-6-chloro-4-hydroxy-2-quinolone-3-carboxamide (**9**)

A mixture of ethyl 6-chloro-4-hydroxy-2-quinolone-3-carboxylate (**5**) (1.00 g, 3.74 mmol) and 3-trifluromethyl aniline (**6c**) (1.80 g, 11.20 mmol) was prepared THF (25 mL). A few drops of DMF were added to the solution and refluxed for 72 h. Reaction progress was monitored by the absence of a spot of **5** on TLC and a precipitate appearance at RT. A solid residue was formed, washed with H_2_O, MeOH, and THF, and then dried in a vacuum oven at 70 °C for 1 h.

A pale yellow powder; yield: 70.9%, R_ƒ_ = 0.75 (CHCl_3_: MeOH- 9.5:0.5); m.p: 313–314 °C; ^1^H-NMR (400 MHz, DMSO-*d*_6+_NaOD): δ = 6.94–6.99 (m, 2H, Ar-*H)*, 7.07 (dd, *J* = 2.8, 2.8 Hz, 1H, Ar-*H*), 7.52 (d, *J* = 8.4 Hz, 1H, Ar-*H*), 7.71–7.81 (m, 2H, Ar-*H*), 7.87 (d, *J* = 8.4 Hz, 1H, Ar-*H*) ppm; ^13^C-NMR (100 MHz, DMSO-*d*_6_): δ = 100.8 (1C), 119.9 (1C), 120.8 (1C), 121.14 (1C), 123.13 (2C), 123.5 (1C), 123.9 (1C), 124.4 (1C), 124.8 (1C), 125.8 (1C), 126.5 (1C), 145.4 (1C), 149.4 (1C), 170.3 (1C), 172.5 (1C), 173.9 (1C), 178.8 (1C) ppm; IR (KBr disc): 3036, 2964, 2902, 1658, 1602, 1467, 1415, 1375, 1325 cm^−1^. High-resolution mass spectrum, *m/z*: calculated 383.04103 for C_17_H_11_ClF_3_N_2_O_3_ [M + H]^+^; found 383.04178.

#### 3.2.5. *N*-(4-(trifluoromethyl)phenyl)-6-chloro-4-hydroxy-2-quinolone-3-carboxamide (10)

A mixture of ethyl 6-chloro-4-hydroxy-2-quinolone-3-carboxylate (**5**) (1.00 g, 3.74 mmol) and 4-trifluromethyl aniline (**6d**) (1.80 g, 11.20 mmol) was prepared in THF (25 mL). A few drops of DMF were added to the solution and refluxed for 72 h. Reaction progress was monitored by the absence of a spot of **5** on TLC and a precipitate appearance at RT. A solid residue was formed, washed with H_2_O, MeOH, and THF, and then dried in a vacuum oven at 70 °C for 1 h.

A white powder; yield: 57.0%, R_ƒ_ = 0.8 (CHCl_3_: MeOH- 9.5:0.5); m.p: 262–264 °C; ^1^H-NMR (400 MHz, DMSO-*d*_6+_NaOD): δ = 6.92–6.95 (m, 1H, Ar-*H*), 7.03–7.05(m, 1H, Ar-*H*), 7.18–7. 21 (m, 1H, Ar-*H*), 7.27–7.35 (m, 2H, Ar-*H)*, 7.76- (m, 1H, Ar-*H*) ppm; ^13^C-NMR (100 MHz, DMSO-*d*_6_): δ = 101.0 (1C), 124.2 (1C), 124.5 (2C), 126.75 (4C), 127.7 (4C), 141.43 (1C), 148.77 (1C), 171.86 (1C), 173.72 (1C), 175.7 (1C) ppm; IR (KBr disc): 3240, 3151, 2980, 1674, 1599, 1469, 1437, 1383, 1329 cm^−1^. High-resolution mass spectrum, *m/z*: calculated 383.04103 for C_17_H_11_ClF_3_N_2_O_3_ [M + H]^+^; found 383.04113.

#### 3.2.6. *N*-(4-methoxyphenyl)-6-chloro-4-hydroxy-2-quinolone-3-carboxamide (**11**)

A mixture of ethyl 6-chloro-4-hydroxy-2-quinolone-3-carboxylate (**5**) (1.00 g, 3.74 mmol) and 4-Anisidine (**6e**) (1.38 g, 11.20 mmol) was prepared in THF (25 mL). A few drops of DMF were added to the solution and refluxed for 72 h. Reaction progress was monitored by the absence of a spot of **5** on TLC and a precipitate appearance at RT. A solid residue was formed, washed with H_2_O, MeOH, and THF, and then dried in a vacuum oven at 70 °C for 1 h.

A brown powder; yield: 75.1%, R_ƒ_ = 0.8 (CHCl_3_: MeOH- 9.5:0.5); m.p: 315–317 °C; ^1^H-NMR (400 MHz, DMSO-*d*_6+_NaOD): δ = 3.7 (s, 3H, OC*H*_3_), 6.81 (d, *J* = 8.8 Hz, 2H, Ar-*H*), 6.94–7. 06 (m, 4H, Ar-*H*), 7.61 (d, *J* = 8.8 Hz, 2H, Ar-*H)*, 7.80 (s, 1H, Ar-*H*) ppm; ^13^C-NMR (100 MHz, DMSO-*d*_6_): δ = 55.5 (1C), 101.1(1C), 114.01 (3C), 119.6 (1C), 121.1 (3C), 124.3 (1C), 124.4 (1C), 135.1 (1C), 149.1 (1C), 154.1 (1C), 169.7 (1C), 173.8 (1C), 175.9 (1C) ppm; IR (KBr disc): 3840, 3730, 1660, 1612, 1566, 1508, 1465, 1417 cm^−1^. High-resolution mass spectrum, *m/z*: calculated 345.06421 for C_17_H_14_ClN_2_O_4_ [M + H]^+^; found 345.06478.

#### 3.2.7. *N*-p-tolyl-6-chloro-4-hydroxy-2-quinolone-3-carboxamide (**12**)

A mixture of ethyl 6-chloro-4-hydroxy-2-quinolone-3-carboxylate (**5**) (1.00 g, 3.74 mmol) and 4-toluidine (**6f**) (1.20 g, 11.20 mmol) was prepared in THF (25 mL). A few drops of DMF were added to the solution and refluxed for 72 h. Reaction progress was monitored by the absence of a spot of **5** on TLC and a precipitate appearance at RT. A solid residue was formed, washed with H_2_O, MeOH, and THF, and then dried in a vacuum oven at 70 °C for 1 h.

An off white powder; yield: 69.5%, R_ƒ_ = 0.8 (CHCl_3_: MeOH- 9.5:0.5); m.p: 333–334 °C; ^1^H-NMR (400 MHz, DMSO-*d*_6+_NaOD): δ = 2.24 (s, 3H, C*H*_3_), 6.93–6.96(m, 1H, Ar-*H*), 7.01–7. 07 (m, 3H, Ar-*H*), 7.59–7.61 (m, 2H, Ar-*H)*, 7.80–7.81 (m, 1H, Ar-*H*), ppm; ^13^C-NMR (100 MHz, DMSO-*d*_6_): δ = 20.9 (1C), 101.1(1C), 124.3 (3C), 124.5 (2C), 128.6 (1C), 129.3 (3C), 129.8 (1C), 139.32 (1C), 149.2 (1C), 169.9 (1C), 173.9 (1C), 176.9 (1C) ppm; IR (KBr disc): 3149, 3020, 2974, 1658, 1602, 1508, 1467, 1415, 1371 cm^−1^. High-resolution mass spectrum, *m/z*: calculated 329.06930 for C_17_H_14_ClN_2_O_3_ [M + H]^+^; found 329.06945.

#### 3.2.8. *N*-(5-chlorobenzoic acid)-6-chloro-4-hydroxy-2-quinolone-3-carboxamide (**13**)

A mixture of ethyl 6-chloro-4-hydroxy-2-quinolone-3-carboxylate (**5**) (1.00 g, 3.74 mmol) and 2-amino 3-chloro benzoic acid (**6g**) (1.90 g, 11.20 mmol) was prepared together in THF (25 mL). A few drops of DMF were added to the solution and refluxed for 72 h. Reaction progress was monitored by the absence of a spot of **5** on TLC and a precipitate appearance at RT. A solid residue was formed, washed with H_2_O, MeOH, and THF, and then dried in a vacuum oven at 70 °C for 1 h.

A pale yellow powder; yield: 86.2%, R_ƒ_ = 0.26 (CHCl_3_: MeOH- 9.5:0.5); m.p: 222–224 °C; ^1^H-NMR (400 MHz, DMSO-*d*_6_): δ =16.27 (s*,* 1H, -*OH* phenol*),* 13.22 (s, 1H, *-COOH*), 12.41 (s, 1H, Ar-*NH*), 12.24 (s, 1H, -*NH* amide), 7.81–7.87 (m, 3H, Ar-*H*), 7.34–7.55 (m, 3H, Ar-*H*) ppm; ^13^C-NMR (100 MHz, DMSO-*d*_6_): δ = 96.9 (1C), 97.5 (1C), 116.5 (1C), 119.4 (1C), 123.7 (1C), 128.2 (1C), 128.4 (1C), 129.3 (1C), 129.4 (1C), 130.7 (1C), 130.8 (1C), 131.3 (1C), 163.0 (1C), 167.14 (1C), 169.7 (1C), 170.0 (1C) ppm; IR (KBr disc): 3482, 3368, 3057, 1667, 1586, 1545, 1458, 1420, 1314 cm^−1^. High-resolution mass spectrum, *m/z*: calculated 393.00450 for C_17_H_11_Cl_2_N_2_O_5_ [M + H]^+^; found 393.00398.

#### 3.2.9. *N*-(3-chlorobenzoic acid)-6-chloro-4-hydroxy-2-quinolone-3-carboxamide (**14**)

A mixture of ethyl 6-chloro-4-hydroxy-2-quinolone-3-carboxylate (**5**) (1.00 g, 3.74 mmol) and 2-amino 5-chloro benzoic acid (**6h**) (1.90 g, 11.20 mmol) was prepared in THF (25mL). A few drops of DMF were added to the solution and refluxed for 72 h. Reaction progress was monitored by the absence of a spot of **5** on TLC and a precipitate appearance at RT. A solid residue was formed, washed with H_2_O, MeOH, and THF, and then dried in a vacuum oven at 70 °C for 1 h.

An off white powder; yield: 97.6%, R_ƒ_ = 0.23 (CHCl_3_: MeOH- 9.5:0.5); m.p: 236–237 °C; ^1^H-NMR (400 MHz, DMSO-*d*_6+_NaOD): δ = 6.56 (d, *J* = 8.4, 2H, Ar-*H),* 6.92–7.06 (m, 1H, Ar-*H*), 7.60–7.67 (m, 2H, Ar-*H*), 7.88 (s, 1H, Ar-*H*), 8.61 (pseudo d, 1H, *-NH-side chain*), 7.97 (d, *J* = 8.8 Hz, 1H, Ar-*H*) ppm; ^13^C-NMR (100 MHz, DMSO-*d*_6_): δ = 117.3 (2C), 117.7 (2C), 122.0 (2C), 129.9 (2C), 131.3 (2C), 138.7 (1C), 149.0 (2C), 170.1 (1C), 171.1 (1C), 173.5 (1C), 175.7 (1C) ppm; IR (KBr disc): 3502, 3470, 3387, 1672, 1585, 1548, 1483, 1419, 1296 cm^−1^. High-resolution mass spectrum, *m/z*: calculated 393.00450 for C_17_H_11_Cl_2_N_2_O_5_ [M + H]^+^; found 393.00489.

#### 3.2.10. *N*-(2-chlorobenzoic acid)-6-chloro-4-hydroxy-2-quinolone-3-carboxamide (**15**)

A mixture of ethyl 6-chloro-4-hydroxy-2-quinolone-3-carboxylate (**5**) (1.00 g, 3.74 mmol) and 2-amino 6-chloro benzoic acid (**6i**) (1.90 g, 11.20 mmol) was prepared in THF (25mL). A few drops of DMF were added to the solution and refluxed for 72 h. Reaction progress was monitored by the absence of a spot of **5** on TLC and a precipitate appearance at RT. A solid residue was formed, washed with H_2_O, MeOH, and THF, and then dried in a vacuum oven at 70 °C for 1 h.

An off white powder; yield: 38.7%, R_ƒ_ = 0.26 (CHCl_3_: MeOH- 8:2); m.p: 310–312 °C; ^1^H-NMR (400 MHz, DMSO-*d*_6_): δ = 7.28 (t, *J* = 8.8 Hz, 1H, Ar-*H),* 7.40–7.53 (m, 1H, Ar-*H*), 7.71 (d, *J* = 2 Hz, 1H, Ar-*H*), 7.76 (d, *J* = 8.4 Hz, 1H, Ar-*H*), 7.83–7.87 (m, 1H, Ar-*H*), 7.91–8.01 (m, 2H, Ar-*H* & *-NH-side chain*), 11.34–11.53 (m, 1H, *-NH-ring*), 12.15 (s, 1H, *-COOH*), 12.64 (s, 1H, *-OH*) ppm; ^13^C-NMR (100 MHz, DMSO-*d*_6_): δ = 115.6 (1C), 122.2 (1C), 123.2 (2C), 123.5 (1C), 124.7 (1C), 125.6 (1C), 126.3 (1C), 126.6 (2C), 127.3 (1C), 128.8 (1C), 138.1 (1C), 138.8 (1C), 161.7 (1C), 162.9 (1C), 163.7 (1C) ppm; IR (KBr disc): 3132, 2980, 2899, 1691, 1645, 1543, 1460, 1373, 1317 cm^−1^. High-resolution mass spectrum, *m/z*: calculated 393.00450 for C_17_H_11_Cl_2_N_2_O_5_ [M + H]^+^; found 393.00425.

#### 3.2.11. *N*-(pyridine-4-yl)-6-chloro-4-hydroxy-2-quinolone-3-carboxamide (**16**)

A mixture of ethyl 6-chloro-4-hydroxy-2-quinolone-3-carboxylate (**5**) (1.00 g, 3.74 mmol) and 4-amino pyridine (**6j**) (1.10 g, 11.20 mmol) was prepared in THF (25 mL). A few drops of DMF were added to the solution and refluxed for 72 h. Reaction progress was monitored by the absence of a spot of **5** on TLC and a precipitate appearance at RT. A solid residue was formed, washed with H_2_O, MeOH, and THF, and then dried in a vacuum oven at 70 °C for 1 h.

A pale pink powder; yield: 50.1%, R_ƒ_ = 0.1 (CHCl_3_: MeOH- 9.5:0.5); m.p: 312–314 °C; ^1^H-NMR (400 MHz, DMSO-*d*_6+_NaOD): δ = 6.98 (d, *J* = 8.8 Hz, 2H, Ar-*H*), 7.08 (dd, *J* = 2.8, 2.8 Hz 2H, Ar-*H*), 7.67 (d, *J* = 3.2 Hz, 2H, Ar-*H)*, 7.81 (s, 1H, Ar-*H*), 8.23 (s, 1H, *-NH-side chain*) ppm; ^13^C-NMR (100 MHz, DMSO-*d*_6_): δ = 100.9 (1C), 114.2 (2C), 120.1 (1C), 124.2 (1C), 124.3 (1C), 124.7 (1C), 129.3 (1C), 148.14 (2C), 149.3 (1C), 150.0 (1C), 170.6 (1C), 173.5 (1C), 176.9 (1C) ppm; IR (KBr disc): 3068, 2987, 2912, 1676, 1583, 1537, 1425, 1377, 1240 cm^−1^. High-resolution mass spectrum, *m/z*: calculated 316.04889 for C_15_H_11_ClN_3_O_3_ [M + H]^+^; found 316.04824.

#### 3.2.12. *N*-(pyridine-3-yl)-6-chloro-4-hydroxy-2-quinolone-3-carboxamide (**17**)

A mixture of ethyl 6-chloro-4-hydroxy-2-quinolone-3-carboxylate (**5**) (1.00 g, 3.74 mmol) and 3-amino pyridine (**6k**) (1.10 g, 11.20 mmol) was put together in THF (25 mL). A few drops of DMF were added to the solution and refluxed for 72 h. Reaction progress was monitored by the absence of a spot of **5** on TLC and a precipitate appearance at RT. A solid residue was formed, washed with H_2_O, MeOH, and THF, and then dried in a vacuum oven at 70 °C for 1 h.

A white powder; yield: 61.5%, R_ƒ_ = 0.56 (CHCl_3_: MeOH- 9.5:0.5); m.p: 347–348 °C; ^1^H-NMR (400 MHz, DMSO-*d*_6+_NaOD): δ = 6.88–6.98 (m, 4H, Ar-*H*), 7.6–7.8 (m, 3H, Ar-*H*), 8.1 (s, 1H, *-NH-side chain*), 8.81 (s, 1H, Ar-*H*) ppm; ^13^C-NMR (100 MHz, DMSO-*d*_6_): δ = 100.78 (2C), 102.4 (2C), 123.97 (1C), 124.04 (1C), 124.31 (1C), 124.4 (1C), 124.6(1C), 125.02 (1C), 128.9 (1C), 138.5 (1C), 141.3 (1C), 172.5 (1C), 172.91 (1C), 173.3 (1C), 176.5 (1C), ppm; IR (KBr disc): 3156, 2992, 2928, 1672, 1609, 1477, 1414, 1377, 1325 cm^−1^. High-resolution mass spectrum, *m/z*: calculated 316.04889 for C_15_H_11_ClN_3_O_3_ [M + H]^+^; found 316.04878.

#### 3.2.13. *N*-(3-benzoic acid)-6-chloro-4-hydroxy-2-quinolone-3 carboxamide (**18**)

A mixture of ethyl 6-chloro-4-hydroxy-2-quinolone-3-carboxylate (**5**) (1.00 g, 3.74 mmol) and 3-amino benzoic acid (**6l**) (1.50 g, 11.20 mmol) was prepared in THF (25 mL). A few drops of DMF were added to the solution and refluxed for 72 h. Reaction progress was monitored by the absence of a spot of **5** on TLC and a precipitate appearance at RT. A solid residue was formed, washed with H_2_O, MeOH, and THF, and then dried in a vacuum oven at 70 °C for 1 h.

A white powder; yield: 44.8%, R_ƒ_ = 0.21 (CHCl_3_: MeOH- 8.0:2.0); m.p: 342–344 °C; ^1^H-NMR (400 MHz, DMSO-*d*_6+_NaOD): δ = 6.96 (d, *J* = 8.4 Hz, 1H, Ar-*H*), 7.07 (dd, *J* = 2.0, 2.4 Hz, 1H, Ar-*H*), 7.14 (t, *J* = 7.6 Hz, 1H, Ar-*H*), 7.45 (d, *J* = 7.6 Hz, 1H, Ar-*H*), 7.78 (d, *J* = 8 Hz, 1H, Ar-*H*), 7.8 (s, 1H, Ar-*H*), = 8.13 (s, 1H, Ar-*H*) ppm; ^13^C-NMR (100 MHz, DMSO-*d*_6_): δ = 101.2 (1C), 119.6 (2C), 120.6 (1C), 121.3 (1C), 122.6 (1C), 124.3 (1C), 124.5 (1C), 127.6 (1C), 128.7 (1C), 140.4 (1C), 140.7 (1C), 149.1 (1C), 169.9 (1C), 171.3(1C), 173.9(1C), 176.0 (1C) ppm; IR (KBr disc): 3037, 2976, 2899, 1691, 1658, 1458, 1419, 1371, 1315 cm^−1^. High-resolution mass spectrum, *m/z*: calculated 359.04347 for C_17_H_12_ClN_2_O_5_ [M + H]^+^; found 359.04301.

#### 3.2.14. *N*-(2-fluorphenyl)-6-chloro-4-hydroxy-2-quinolone-3-carboxamide (**19**)

A mixture of ethyl 6-chloro-4-hydroxy-2-quinolone-3-carboxylate (**5**) (1.00 g, 3.74 mmol) and 2-fluoroaniline (**6m**) (1.20 g, 11.20 mmol) was prepared in THF (25 mL). A few drops of DMF were added to the solution and refluxed for 72 h. Reaction progress was monitored by the absence of a spot of **5** on TLC and a precipitate appearance at RT. A solid residue was formed, washed with H_2_O, MeOH, and THF, and then dried in a vacuum oven at 70 °C for 1 h.

An off white powder; yield: 72.4%, R_ƒ_ = 0.7 (CHCl_3_: MeOH- 9.5:0.5); m.p: 318–320 °C; ^1^H-NMR (400 MHz, DMSO-*d*_6+_NaOD): δ = 6.85–6.90(m, 1H, Ar-*H*), 6.93–7.03 (m, 3H, Ar-*H*), 7.05–7.12 (m, 1H, Ar-*H)*, 7.8 (s, 1H, Ar-*H*), 8.68 (t, *J* = 8 Hz, 1H, Ar-*H*) ppm; ^13^C-NMR (100 MHz, DMSO-*d*_6_): δ = 101.0 (1C), 119.5(1C), 121.1 (2C), 121.2 (1C), 122.1 (1C), 124.4 (2C), 124.5 (1C), 124.6 (1C), 149.5 (1C), 151.4 (1C), 153.8 (1C), 170.2 (1C), 174.0 (1C), 176.6 (1C) ppm; IR (KBr disc): 2980, 2902, 2833, 1666, 1604, 1566, 1469,1417, 1373 cm^-1^. High-resolution mass spectrum, *m/z*: calculated 333.04422 for C_16_H_11_ClFN_2_O_3_ [M + H]^+^; found 333.04465.

#### 3.2.15. *N*-(3-fluorphenyl)-6-chloro-4-hydroxy-2-quinolone-3-carboxamide (**20**)

A mixture of ethyl 6-chloro-4-hydroxy-2-quinolone-3-carboxylate (**5**) (1.00 g, 3.74 mmol) and 3-fluoroaniline (**6n**) (1.20 g, 11.20 mmol) was prepared in THF (25 mL). A few drops of DMF were added to the solution and refluxed for 72 h. Reaction progress was monitored by the absence of a spot of **5** on TLC and a precipitate appearance at RT. A solid residue was formed, washed with H_2_O, MeOH, and THF, and then dried in a vacuum oven at 70 °C for 1 h.

An off white powder; yield: 64.4%, R_ƒ_ = 0.75 (CHCl_3_: MeOH- 9.5:0.5); m.p: 298–300 °C; ^1^H-NMR (400 MHz, DMSO-*d*_6+_NaOD): δ = 6.68 (t, *J* = 8.0, 1H, Ar-*H),* 7.16–7.24 (m, 2H, Ar-*H*), 7.80 (s, 1H, Ar-*H*), 7.95 (pseudo d, 1H, Ar-*H*), 7.97 (d, *J* = 8.8 Hz, 1H, Ar-*H*) ppm; ^13^C-NMR (100 MHz, DMSO-*d*_6_): δ = 100.95 (1C), 106.5 (1C), 107.3 (1C), 115.3 (1C), 119.9 (1C), 124.3 (2C), 128.9 (1C), 130.1 (1C), 130.2 (1C), 143.5 (1C), 149.2 (1C), 161.7 (1C), 164.1 (1C), 170.1 (1C), 173.5 (1C), 176.4 (1C) ppm; IR (KBr disc): 2972, 2899, 2829, 1658, 1604, 1493, 1415, 1554, 1373, 1328 cm^−1^. High-resolution mass spectrum, *m/z*: calculated 333.04422 for C_16_H_11_ClFN_2_O_3_ [M + H]^+^; found 333.04411.

#### 3.2.16. *N*-(4-fluorphenyl)-6-chloro-4-hydroxy-2-quinolone-3-carboxamide (**21**)

A mixture of ethyl 6-chloro-4-hydroxy-2-quinolone-3-carboxylate (**5**) (1.00 g, 3.74 mmol) and 4-fluoroaniline (**6o**) (1.20 g, 11.20 mmol) was prepared in THF (25 mL). A few drops of DMF were added to the solution and refluxed for 72 h. Reaction progress was monitored by the absence of a spot of **5** on TLC and a precipitate appearance at RT. A solid residue was formed, washed with H_2_O, MeOH, and THF, and then dried in a vacuum oven at 70 °C for 1 h.

An off white powder; yield: 80.5%, R_ƒ_ = 0.75 (CHCl_3_: MeOH- 9.5:0.5); m.p: 300–302 °C; ^1^H-NMR (400 MHz, DMSO-*d*_6+_NaOD): δ = 6.95–7.03 (m, 3H, Ar-*H*), 7.06 (dd, *J* = 2.4, 2.4 Hz, 1H, Ar-*H*), 7.68–7.71 (m, 2H, Ar-*H*), 7.81 (d, *J* = 2.8 Hz, 1H, Ar-*H*) ppm; ^13^C-NMR (100 MHz, DMSO-*d*_6_): δ = 101.1 (1C), 115.1 (2C), 119.9 (1C), 121.2 (2C), 121.3 (2C), 124.2 (1C), 138.0 (1C), 149.0 (1C), 156.1 (1C), 158.4 (1C), 169.8 (1C), 173.6 (1C), 176.1 (1C) ppm; IR (KBr disc): 3045, 2972, 2902, 1662, 1614, 1506, 1467, 1415, 1371 cm^−1^. High-resolution mass spectrum, *m/z*: calculated 333.04422 for C_16_H_11_ClFN_2_O_3_ [M + H]^+^; found 333.04479.

#### 3.2.17. *N*-(6-methylbenzoic acid)-6-chloro-4-hydroxy-2-quinolone-3-carboxamide (**22**)

A mixture of ethyl 6-chloro-4-hydroxy-2-quinolone-3-carboxylate (**5**) (1.00 g, 3.74 mmol) and 2-amino-6-methyl benzoic acid (**6p**) (1.70 g, 11.20 mmol) was prepared in THF (25 mL). A few drops of DMF were added to the solution and refluxed for 72 h. Reaction progress was monitored by the absence of a spot of **5** on TLC and a precipitate appearance at RT. A solid residue was formed, washed with H_2_O, MeOH, and THF, and then dried in a vacuum oven at 70 °C for 1 h.

An off white powder; yield: 79%, R_ƒ_ = 0.56 (CHCl_3_: MeOH- 9.5:0.5); m.p: 284–285 °C; ^1^H-NMR (400 MHz, DMSO-*d*_6+_NaOD): δ = 2.23 (s, 1H, -C*H_3_*), 6.67 (d, *J* = 7.2 Hz, 1H, Ar-*H*), 6.89 (t, *J* = 8 Hz, 1H, Ar-*H*), 6.99 (d, *J* = 8.8 Hz, 1H, Ar-*H*), 7.05 (d, *J* = 7.2 Hz, 1H, Ar-*H)*, 7.89 (s, 1H, Ar-*H*), 8.23 (d, *J* = 8 Hz, 1H, Ar-*H*) ppm; ^13^C-NMR (100 MHz, DMSO-*d*_6_): δ = 20.3 (1C), 101.8 (1C), 119.1 (1C), 120.03 (1C), 122.7 (1C), 124.5 (1C), 124.7 (1C), 125.03 (1C), 125.4 (2C), 132.0 (1C), 135.7 (1C), 136.2 (1C)), 148.9 (1C), 169.8 (1C), 173.9 (1C), 174.7 (1C),175.8 (1C) ppm; IR (KBr disc): 3482, 3127, 2965, 1680, 1612, 1549, 1470, 1418, 1377 cm^−1^. High-resolution mass spectrum, *m/z*: calculated 373.05912 for C_18_H_14_ClN_2_O_5_ [M + H]^+^; found 373.05945.

#### 3.2.18. *N*-(5-methylbenzoic acid)-6-chloro-4-hydroxy-2-quinolone-3-carboxamide (**23**)

A mixture of ethyl 6-chloro-4-hydroxy-2-quinolone-3-carboxylate (**5**) (1.00 g, 3.74 mmol) and 2-amino-5-methyl benzoic acid (**6q**) (1.70 g, 11.20 mmol) was prepared in THF (25 mL). A few drops of DMF were added to the solution and refluxed for 72 h. Reaction progress was monitored by the absence of a spot of **5** on TLC and a precipitate appearance at RT. A solid residue was formed, washed with H_2_O, MeOH, and THF, and then dried in a vacuum oven at 70 °C for 1 h.

An off white powder; yield: 71.8%, R_ƒ_ = 0.26 (CHCl_3_: MeOH- 8.0:2.0); m.p: 318–320 °C; ^1^H-NMR (400 MHz, DMSO-*d*_6+_NaOD): δ = 2.22 (s, 3H, C*H*_3_), 6.91–6.93(m, 2H, Ar-*H*), 6.99–7. 01 (m, 1H, Ar-*H*), 7.53 (s, 1H, Ar-*H)*, 7.90 (s, 1H, Ar-*H*), 8.50 (d, *J* = 8 Hz, 1H, Ar-*H*) ppm; ^13^C-NMR (100 MHz, DMSO-*d*_6_): δ = 20.9 (1C), 102.6(1C), 119.4 (2C), 122.2 (1C), 124.5 (1C), 124.6 (1C), 128.3 (2C), 128.4 (1C), 125.5 (2C), 129.8 (1C), 149.4 (1C), 170.1 (1C)), 172.9 (1C)), 174.1 (1C)), 175.6 (1C) ppm; IR (KBr disc): 3128, 2949, 2891, 1658, 1616, 1579, 1529, 1460, 1436 cm^−1^. High-resolution mass spectrum, *m/z*: calculated 373.05912 for C_18_H_14_ClN_2_O_5_ [M + H]^+^; found 373.05978.

#### 3.2.19. *N*-(4-methoxybenzoic acid)-6-chloro-4-hydroxy-2-quinolone-3-carboxamide (**24**)

A mixture of ethyl 6-chloro-4-hydroxy-2-quinolone-3-carboxylate (**5**) (1.00 g, 3.74 mmol) and 2-amino-4-methoxy benzoic acid (**6r**) (1.90 g, 11.20 mmol) was prepared in THF (25 mL). A few drops of DMF were added to the solution and refluxed for 72 h. Reaction progress was monitored by the absence of a spot of **5** on TLC and a precipitate appearance at RT. A solid residue was formed, washed with H_2_O, MeOH, and THF, and then dried in a vacuum oven at 70 °C for 1 h.

A white powder; yield: 82.6%, R_ƒ_ = 0.13 (CHCl_3_: MeOH- 8.0:2.0); m.p: 273–274 °C; ^1^H-NMR (400 MHz, DMSO-*d*_6+_NaOD): δ = 3.73 (s, 3H, C*H_3_*), 6.37 (dd, *J* = 2.4, 2.4 Hz, 1H, Ar-*H*), 6.92 (d, *J* = 8.8 Hz, 1H, Ar-*H*), 7.01–7.03 (m*,* 1H, Ar-*H*), 7.69 (d, *J* = 8.4 Hz, 1H, Ar-*H*), 7.87 (d, *J* = 4 Hz, 1H, Ar-*H*), 8.39 (s*,* 1H, Ar-*H*) ppm; ^13^C-NMR (100 MHz, DMSO-*d*_6_): δ = 55.26 (1C), 103.2 (1C), 105.7 (1C), 106.7 (2C), 119.5 (1C), 121.7 (2C), 124.3 (1C), 124.5 (1C), 125.3 (1C), 131.9 (1C), 142.0 (1C), 149.2 (1C), 159.9 (1C), 170.5 (1C), 172.2 (1C), 173.5 (1C), 175.2(1C) ppm; IR (KBr disc): 3124, 2920, 2845, 1676, 1616, 1544, 1446, 1377, 1261 cm^−1^. High-resolution mass spectrum, *m/z*: calculated 389.05404 for C_18_H_14_ClN_2_O_6_ [M + H]^+^; found 389.05425.

### 3.3. Biology

#### 3.3.1. Culture Conditions

Two human colon cancer cell lines—Caco-2 and HCT-116—were grown in DMEM culture medium (Dulbecco’s modified essential medium, Gibco), complemented with 5% (*v/v*) fetal calf serum (JS Bioscience, Melbourne, Australia), and 1% (*v/v*) antibiotic (2 mM L-glutamine, 100 U/mL Penicillin and 0.1 mg/mL Streptomycin; Gibco). Cells were cultured at 37 °C in a humidified 5% CO_2_ incubator. Enzymatic detachment of the confluent cell layers was carried out using Trypsin/EDTA (Gibco, Gaithersburg, MD, USA). To assess cell viability, trypan blue vital staining (0.4% (*w/v*); Sigma, New York, NY, USA) was employed with cell number, which was evaluated using a light microscope.

#### 3.3.2. MTT Assay

All cells were plated at a density of 8 × 10^3^ cells per well in 96-well plates and incubated to allow attachment for 24 h. The in vitro evaluation of the antiproliferative activities of the examined series was accomplished using the 3-(4,5-dimethylthiazol-2yl)-2,5-diphenyl tetrazolium bromide (MTT) colorimetric assay, as previously described [42]. In short, compounds were diluted in culture media to yield the required concentration and applied to test wells for 48 h at 37 °C in a 5% CO_2_ incubator. Three triplicates of each concentration for all tested compounds were conducted in three independent assays (*n* = 9). DMEM samples were employed as negative controls, and LY294002 as a positive control. At the end of the exposure period, 20 µL of 0.5 mg/mL of MTT was added to each well and incubated for 4 h, afterword its reduction to formazan by metabolically active cells was calculated via measuring the absorbance at 570 nm. Cell viability was calculated based on the measured absorbance relative to the absorbance of the cells exposed to the negative control, which represented 100% cell viability.

#### 3.3.3. Statistical Analysis

Data analysis was performed using GraphPad Prism software version 7. The differences between treatments groups were determined by t-test or one-way analysis of variance (ANOVA) followed by Tukey post hoc t-test as appropriate. Data were expressed as mean ± SD and *p* < 0.05 was considered a statistically significant difference. A non-linear regression analysis was used to calculate IC_50_ values.

#### 3.3.4. Quantitative Real-Time PCR

##### RNA Extraction

Total RNA was extracted from the cultured cells using Quick-RNA MiniPrep according to the manufacturer’s instructions as follows: harvested cells were re-suspended in RNA lysis buffer and centrifuged, the supernatant then was transferred into a Spin-Away Filter in a collection tube and centrifuged, and then 95% ethanol was added and mixed well. The mixture was then transferred to a Zymo-Spin IIICG Column in a collection tube and centrifuged, followed by adding RNA Prep Buffer to the column, followed by two washing steps by adding RNA wash buffer to the column and then centrifugation. Finally, the RNA was eluted by adding DNase/RNase-free water to the column and centrifuged. Samples were quantified using a spectrophotometer via absorbance at 260/280 nm.

##### Complementary DNA (cDNA) Synthesis

Complementary cDNA synthesis was performed using a ProtoScript First Strand cDNA Synthesis Kit following the manufacturer’s instructions. In brief, 1000 nanogram from total RNA was add to a total 20 µL reaction volume that included 60 µM random primer mix, reaction mix, 10 × enzyme mix, and nuclease-free water. The tubes were then incubated at 25 °C for 5 min followed by incubation at 42 °C for 1 h. The cDNA was then then stored at −20 °C until further analysis.

##### Real-Time PCR

The sequences of the primers that were used in real-time PCR assay are shown in Table 4. The reaction mixtures consisted of 200 ng cDNA template, 10 µM of each primer, 10 µL of 2X SYBER Premix Ex Taq II (Takara BIO INC, Shiga, Japan), and the total reaction volume was 20 µL. The reaction was carried out using a BIO RAD iQ5 Multicolor real-time PCR Detection System thermal cycler under the following reaction conditions: 1 cycle of 2 min at 95.0 °C, followed by 45 cycles of 10 s at 95.0 °C, 25 s at 57.0 °C, 25 s at 60.0 °C, and a final cycle of 30 s at 55.0 °C. To confirm that only one PCR product was amplified, dissociation curve analysis of amplification products was performed at the end of the last amplification cycle.

### 3.4. Computational Methods

#### 3.4.1. Preparation of PI3Kα Structure

The co-ordinates of WT PI3Kαs (PDB ID: 2RD0) [9] and (PDB ID: 4L23) [39] as well as MUT (H1047R) PI3Kα (PDB ID: 3HHM) [35] were obtained from the RCSB Protein Data Bank [3,4,5,6,7,8,9,10,11,12,13,14,15,16,17,18,19,20,21,22,23,24,25,26,27,28,29,30,31,32,33,34,35,36,37,38,39,40,41,42,43,44,45]. The template of X6K in 4L23 [39] was conveyed to 2RD0 and pinpointed as a ligand to extract the grid file. The homology modeled assemblies of 2RD0 and 3HHM were previously reported by our research group [19] and employed for this study. The co-ordinates of 2RD0 and 3HHM were energetically minimized to avoid steric clash. Extra treatment for the minimized co-ordinates was executed using Protein Preparation algorithm in Schrödinger software [38] to maximize H-bond interactions between side chains.

#### 3.4.2. Preparation of Ligand Structures

The verified compounds (ligands) were modeled using wortmannin’s template in 3HHM. The ligands were built by MAESTRO [38] Build script and energetically minimized by the MacroModel algorithm using OPLS2005 force field.

#### 3.4.3. Induced-Fit Docking (IFD)

The adopted ligand (wortmannin) was labeled as a centroid in 2RD0 [9] and 3HMM [35] kinase domains. The Van der Waals scaling factors for receptor and ligand were calibrated to 0.5 to provide adequate flexibility for the best docked ligand pose. Other parameters were adjusted as default. The ligand geometry with the highest XP Glide binding affinity was recorded.

#### 3.4.4. Molecular Descriptors

All molecular structures, sketched in ChemDraw [46] and saved in SDF file format, were standardized according to the methods described by Hajjo et al. [47]. Next, two groups of molecular descriptors, comprising ‘Drug-like Indices’ and ‘Molecular Properties’ calculated using alvaDesc software from Kode Cheminformatics [48], were generated for compounds, **5**,**7**–**27**.

#### 3.4.5. Principal Component Analysis (PCA)

A principal component analysis was performed on structures **5** and **7**–**27** using drug-like indices and molecular properties. All calculations and PCA analysis were performed using alvaDesc software from Kode Cheminformatics [48].

## 4. Conclusions

The phosphatidylinositol 3-kinase (PI3Kα) has been recognized as a “hot” target for anticancer drug design. We disclosed a library of *N*-phenyl-6-chloro-4-hydroxy-2-quinolone-3-carboxamides as potential PI3Kα inhibitors.

Biological investigation in Caco-2 and HCT116 cell lines showed potent suppression HCT-116 mediated by compounds (**16**, **18**, **19**, **21**, and **24**). IFD studies against PI3Kαs illustrated that the series accommodate PI3Kαs binding sites and engage with key residues involved in inhibitor interactions. We are looking for optimizing the core structure of this series to induce its anticancer activity and selectivity against a panel of kinases.

## Figures and Tables

**Table 1:** Structures of synthesized derivatives of *N*-phenyl-6-chloro-4-hydroxy- 2-quinolone- 3- carboxamide (**7**–**24**).

**Compound**	**Ar**	**Compound**	**Ar**
**7**	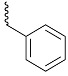	**16**	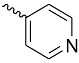
**8**	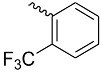	**17**	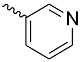
**9**	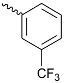	**18**	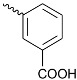
**10**	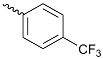	**19**	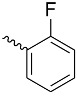
**11**	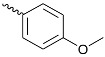	**20**	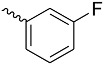
**12**	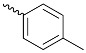	**21**	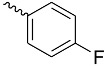
**13**	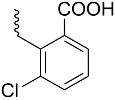	**22**	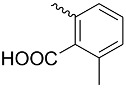
**14**	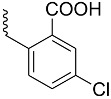	**23**	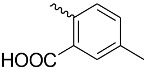
**15**	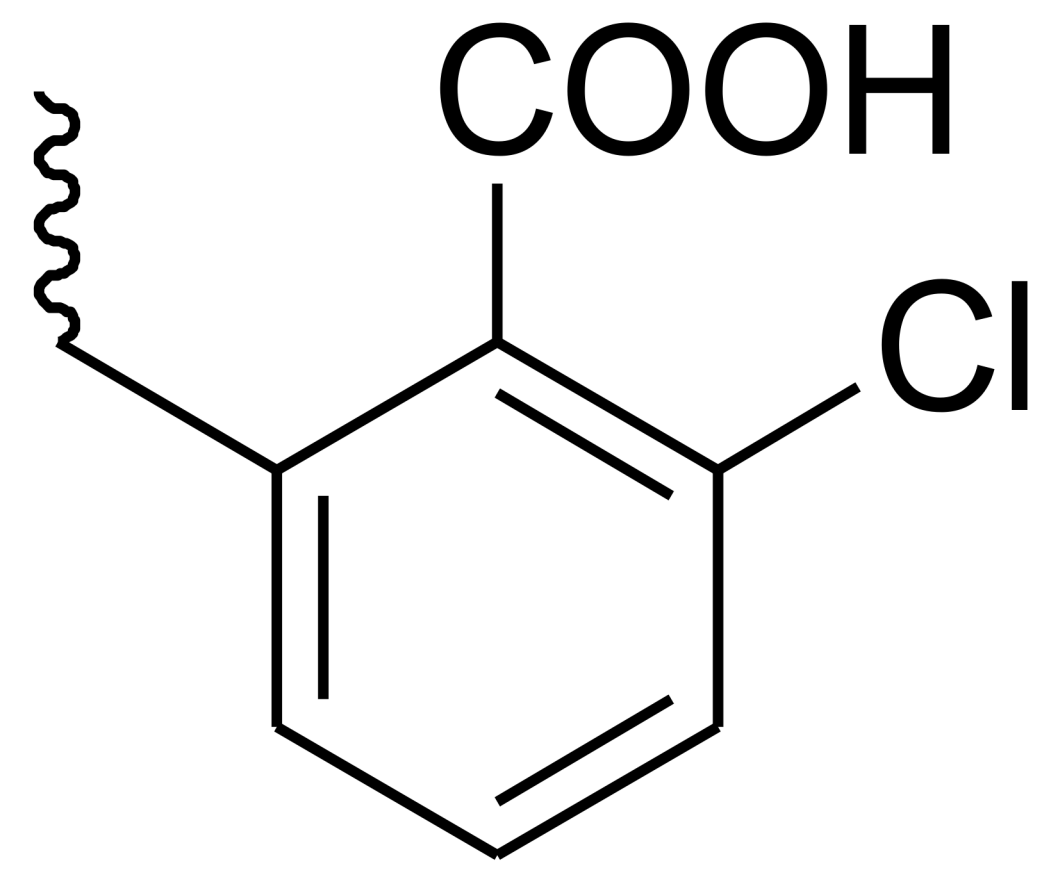	**24**	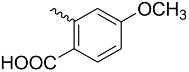

**Table 2:** Inhibitory activity (IC_50_ µM) of **5**, **7–24**.

Compound	IC_50_ (µM) ± SD		Compound	IC_50_ (µM) ± SD	
	Caco-2	HCT-116			
**LY294002**	7.4	6.5	**15**	61.0 ± 4	79.5 ± 3
**5**	75.1 ± 8	78.4 ± 9	**16**	37.4 ± 3	8.9 ± 1
**7**	13.8 ± 1	14.5 ± 2	**17**	35.8 ± 4	33.2 ± 6
**8**	17.9 ± 4	14.5 ± 2	**18**	50.9 ± 3	3.3 ± 0.2
**9**	20.3 ± 2	28.9 ± 2	**19**	17.0 ± 4	5.3 ± 1
**10**	29.7 ± 4	37.0 ± 6	**20**	16.4 ± 3	27.0 ± 5
**11**	14.1 ± 1	9.3 ± 1	**21**	18.9 ± 2	4.9 ± 1
**12**	54.1 ± 5	56.3 ± 5	**22**	31.4 ± 3	50.2 ± 6
**13**	81.4 ± 6	51.9 ± 3	**23**	66.5 ± 7	47.0 ± 3
**14**	14.1 ± 1	16.5 ± 1	**24**	23.0 ± 2	10.5 ± 1

**Table 3:** Induced-fit docking (IFD) scores (Kcal/mol) and H-bond interactions against wild-type (WT) (2RD0) and mutant (MUT) (3HHM) PI3Kα.

Compound	2RD0		3HHM	
	Docking Score	Binding Residues	Docking Score	Binding Residues
**5**	−8.11	K802, Y836, D933	−7.89	W780, Y836, E849, D933
**7**	−8.38	E849, V851, S854	−7.19	S774, D933
**8**	−9.01	V851	−8.16	S774, D933
**9**	−8.99	V851	−8.37	N920
**10**	−8.74	V851, S854	−9.09	Y836, D933
**11**	−9.00	K802, V851	−8.74	Y836, V851
**12**	−8.74	V851, S854	−10.73	E849, V851, D933
**13**	−8.94	E849, V851	−8.32	S774, A775, D933
**14**	−9.29	K802, D933	−8.20	S774, D933
**15**	−7.65	V851, Q859, E798	−9.73	S774, A775, K776, D933
**16**	−7.56	V851	−7.89	S774, S919, D933
**17**	−8.83	V851	−8.56	S774, D933, N920
**18**	−10.50	K802, D810, Y836, V851, S854, D933	−9.36	S774, D933, N920
**19**	−8.44	V851, Q859	−7.89	S774, D933, N920
**20**	−7.86	V851	−8.47	S774, D933, N920
**21**	−8.46	V851	−8.18	S774, D933, N920
**22**	−9.05	S854, Q859	−10.41	S774, A775, K776, D933
**23**	−8.60	V851, S854	−9.20	E849, Y836, D933
**24**	−10.33	V851, N853, S854, Q859	−9.02	S774, Y836, D933

**Table 4:** Sequences of primers that were used to detect the expression of β-actin, BAD, PI3K, and AKT.

Target	Forward Primers (5′ → 3′)	Reverse Primers (5′ → 3′)
β-actin	ACGGGGTCACCCACACTGTGC	CTAGAAGCATTTGCGGTGGACGATG
BAD	CCTCAGGCCTATGCAAAAAG	AAACCCAAAACTTCCGATGG
PI3K	ACCCAGCAACAGAAAAATGG	GCGCTGTGAATTTAGCCTTC
AKT	AACCTGTGCTCCATGACCTC	CCCTTCTACAACCAGGACCA

## Supplementary Materials

The following are available online: Table S1: Calculated descriptors of the synthesized compounds (**5**, **7–24**).

## Sample Availability

Samples of the compounds are available from the authors.
